# Statistical and Machine Learning Techniques in Human Microbiome Studies: Contemporary Challenges and Solutions

**DOI:** 10.3389/fmicb.2021.635781

**Published:** 2021-02-22

**Authors:** Isabel Moreno-Indias, Leo Lahti, Miroslava Nedyalkova, Ilze Elbere, Gennady Roshchupkin, Muhamed Adilovic, Onder Aydemir, Burcu Bakir-Gungor, Enrique Carrillo-de Santa Pau, Domenica D’Elia, Mahesh S. Desai, Laurent Falquet, Aycan Gundogdu, Karel Hron, Thomas Klammsteiner, Marta B. Lopes, Laura Judith Marcos-Zambrano, Cláudia Marques, Michael Mason, Patrick May, Lejla Pašić, Gianvito Pio, Sándor Pongor, Vasilis J. Promponas, Piotr Przymus, Julio Saez-Rodriguez, Alexia Sampri, Rajesh Shigdel, Blaz Stres, Ramona Suharoschi, Jaak Truu, Ciprian-Octavian Truică, Baiba Vilne, Dimitrios Vlachakis, Ercument Yilmaz, Georg Zeller, Aldert L. Zomer, David Gómez-Cabrero, Marcus J. Claesson

**Affiliations:** ^1^Instituto de Investigación Biomédica de Málaga (IBIMA), Unidad de Gestión Clìnica de Endocrinologìa y Nutrición, Hospital Clìnico Universitario Virgen de la Victoria, Universidad de Málaga, Málaga, Spain; ^2^Centro de Investigación Biomeìdica en Red de Fisiopatologtìa de la Obesidad y la Nutrición (CIBEROBN), Instituto de Salud Carlos III, Madrid, Spain; ^3^Department of Computing, University of Turku, Turku, Finland; ^4^Human Genetics and Disease Mechanisms, Latvian Biomedical Research and Study Centre, Riga, Latvia; ^5^Latvian Biomedical Research and Study Centre, Riga, Latvia; ^6^Department of Epidemiology, Erasmus Medical Center, Rotterdam, Netherlands; ^7^Department of Genetics and Bioengineering, International University of Sarajevo, Sarajevo, Bosnia and Herzegovina; ^8^Department of Electrical and Electronics Engineering, Karadeniz Technical University, Trabzon, Turkey; ^9^Department of Computer Engineering, Abdullah Gul University, Kayseri, Turkey; ^10^Computational Biology Group, Precision Nutrition and Cancer Research Program, IMDEA Food Institute, Madrid, Spain; ^11^Department for Biomedical Sciences, Institute for Biomedical Technologies, National Research Council, Bari, Italy; ^12^Department of Infection and Immunity, Luxembourg Institute of Health, Esch-sur-Alzette, Luxembourg; ^13^Odense Research Center for Anaphylaxis, Department of Dermatology and Allergy Center, Odense University Hospital, University of Southern Denmark, Odense, Denmark; ^14^Department of Biology, University of Fribourg, Fribourg, Switzerland; ^15^Swiss Institute of Bioinformatics, Lausanne, Switzerland; ^16^Department of Microbiology and Clinical Microbiology, Faculty of Medicine, Erciyes University, Kayseri, Turkey; ^17^Metagenomics Laboratory, Genome and Stem Cell Center (GenKök), Erciyes University, Kayseri, Turkey; ^18^Department of Mathematical Analysis and Applications of Mathematics, Palacký University, Olomouc, Czechia; ^19^Department of Microbiology, University of Innsbruck, Innsbruck, Austria; ^20^NOVA Laboratory for Computer Science and Informatics (NOVA LINCS), FCT, UNL, Caparica, Portugal; ^21^Centro de Matemática e Aplicações (CMA), FCT, UNL, Caparica, Portugal; ^22^CINTESIS, NOVA Medical School, NMS, Universidade Nova de Lisboa, Lisbon, Portugal; ^23^Computational Oncology, Sage Bionetworks, Seattle, WA, United States; ^24^Bioinformatics Core, Luxembourg Centre for Systems Biomedicine, University of Luxembourg, Esch-sur-Alzette, Luxembourg; ^25^Sarajevo Medical School, University Sarajevo School of Science and Technology, Sarajevo, Bosnia and Herzegovina; ^26^Department of Computer Science, University of Bari Aldo Moro, Bari, Italy; ^27^Faculty of Information Tehnology and Bionics, Pázmány University, Budapest, Hungary; ^28^Bioinformatics Research Laboratory, Department of Biological Sciences, University of Cyprus, Nicosia, Cyprus; ^29^Faculty of Mathematics and Computer Science, Nicolaus Copernicus University, Toruñ, Poland; ^30^Institute of Computational Biomedicine, Heidelberg University, Faculty of Medicine and Heidelberg University Hospital, Heidelberg, Germany; ^31^Division of Informatics, Imaging and Data Sciences, School of Health Sciences, University of Manchester, Manchester, United Kingdom; ^32^Department of Clinical Science, University of Bergen, Bergen, Norway; ^33^Jozef Stefan Institute, Ljubljana, Slovenia; ^34^Biotechnical Faculty, University of Ljubljana, Ljubljana, Slovenia; ^35^Faculty of Civil and Geodetic Engineering, University of Ljubljana, Ljubljana, Slovenia; ^36^Molecular Nutrition and Proteomics Lab, Faculty of the Food Science and Technology, Institute of Life Sciences, University of Agricultural Sciences and Veterinary Medicine of Cluj-Napoca, Cluj-Napoca, Romania; ^37^Institute of Molecular and Cell Biology, University of Tartu, Tartu, Estonia; ^38^Department of Computer Science and Engineering, Faculty of Automatic Control and Computers, University Politehnica of Bucharest, Bucharest, Romania; ^39^Bioinformatics Research Unit, Riga Stradins University, Riga, Latvia; ^40^Laboratory of Genetics, Department of Biotechnology, School of Applied Biology and Biotechnology, Agricultural University of Athens, Athens, Greece; ^41^Department of Computer Technologies, Karadeniz Technical University, Trabzon, Turkey; ^42^European Molecular Biology Laboratory, Structural and Computational Biology Unit, Heidelberg, Germany; ^43^Department of Infectious Diseases and Immunology, Faculty of Veterinary Medicine, Utrecht University, Utrecht, Netherlands; ^44^Navarrabiomed, Complejo Hospitalario de Navarra (CHN), IdiSNA, Universidad Pública de Navarra (UPNA), Pamplona, Spain; ^45^School of Microbiology and APC Microbiome Ireland, University College Cork, Cork, Ireland

**Keywords:** machine learning, microbiome, ML4Microbiome, personalized medicine, biomarker identification

## Abstract

The human microbiome has emerged as a central research topic in human biology and biomedicine. Current microbiome studies generate high-throughput omics data across different body sites, populations, and life stages. Many of the challenges in microbiome research are similar to other high-throughput studies, the quantitative analyses need to address the heterogeneity of data, specific statistical properties, and the remarkable variation in microbiome composition across individuals and body sites. This has led to a broad spectrum of statistical and machine learning challenges that range from study design, data processing, and standardization to analysis, modeling, cross-study comparison, prediction, data science ecosystems, and reproducible reporting. Nevertheless, although many statistics and machine learning approaches and tools have been developed, new techniques are needed to deal with emerging applications and the vast heterogeneity of microbiome data. We review and discuss emerging applications of statistical and machine learning techniques in human microbiome studies and introduce the COST Action CA18131 “ML4Microbiome” that brings together microbiome researchers and machine learning experts to address current challenges such as standardization of analysis pipelines for reproducibility of data analysis results, benchmarking, improvement, or development of existing and new tools and ontologies.

## Introduction

The microbiome has long been defined as a community of commensal, symbiotic, or pathogenic microorganisms that inhabit a particular body site or environment (Lederberg and McCray, 2001). The current apprehension of the microbiome encompasses the totality of microorganisms and their interactions, interplay with the host and the surrounding environment, and is further influenced by constant co-evolution (Berg et al., 2020). Understanding the composition, balance, and role of the microbiome in human health and disease has become a field of extensive research over the past decade (Wang and Kasper, 2014; Gagnière et al., 2016; Sampson et al., 2016; Barratt et al., 2017). The potential for applications in biomedicine and biotechnology has been especially evident from gut microbiome studies. Furthermore, microbiome research has become an important subject of popular science and led to the acceleration of development in different biotechnology industry sectors.

Some of the key topics in this field cover early life (Tamburini et al., 2016), mechanisms of colonization resistance against pathogens (Buffie and Pamer, 2013; Kim et al., 2017), and stability and individuality of adult microbiota (Mehta et al., 2018), and its associations with diseases, diet, medication, and lifestyle in various populations across the globe (Segata et al., 2011; Schmidt et al., 2018; Cullen et al., 2020). Moreover, the research focus is shifting toward considering the role of genetics and environment (Org et al., 2015; Roslund et al., 2020), as well as of diet (Singh et al., 2017), and to translate this knowledge into microbiota-based clinical solutions (Lynch et al., 2019).

Compared to many other fields of multi-omic studies, microbiomes are dynamic ecosystems with active host regulation. This adds interesting new dimensions and complexity to the analyses and interpretation of data. Thus, the field also requires additional ecological perspectives. The advances in high-throughput sequencing technologies have accelerated microbiome research (Malla et al., 2019), but the volume of data and their complexity sets challenges for analysis. Adaptive statistical and machine learning (ML) methodologies can help us to overcome many of these barriers, but these methodologies need to be adjusted to the specific properties of microbiome data.

### Microbiome Data Properties and Analysis Challenges

Two commonly used strategies for microbiome profiling include the sequencing of a highly conserved region, such as the bacterial 16S ribosomal RNA (16S rRNA), and the untargeted sequencing of genetic material present in the sample, as in shotgun metagenomics (see Box 1 for more information) (Nayfach et al., 2019). The quality of microbiome data and profiling is influenced by experimental, biological, and environmental factors (Poussin et al., 2018). Further variation arises from differences in sequence filtering, clustering, taxonomic assignment and binning, as different bioinformatic tools and pipelines are in use. This lack of standardization introduces statistical biases, and subsequent challenges for reproducibility and cross-study comparisons (Lozupone et al., 2013; Falony et al., 2016; Zhernakova et al., 2016). Some of the first large microbiome profiling studies, as the Human Microbiome Project (Turnbaugh et al., 2007) and the MetaHIT project (Qin et al., 2010), were established as a population-scale framework to develop metagenomic protocols (for a more comprehensive list of large-scale microbiome studies, see Marcos-Zambrano et al., 2021). Despite various attempts to standardize methods, a gold standard of microbiome research is yet to be established (Quince et al., 2017; Knight et al., 2018).

**Box 1. Common data types in microbiome research.**

**Amplicon data**. Amplicon based approaches are the most widely used high-throughput method for microbiome studies. Amplicon studies comprise data from specific regions of various types of marker genes used for taxonomic profile determination of microbiome: 16S ribosomal RNA (16S rRNA) gene for prokaryotes; 18S ribosomal RNA (18S rRNA) gene for eukaryotes; internal transcribed spacers (ITS) for fungi. These data are characterized by variability in the selected regions, amplification primers and amplification protocols. Due to the sequence similarity, the data are often organized into operational taxonomic units (OTUs) (Schmitt et al., 2012). The two most popular approaches for obtaining groups of related OTUs are based on (i) aligning sequences to a reference database or (ii) clustering sequences based on sequence identity (*de novo* approach). Once OTU clusters are defined, taxonomic information is given for the representative sequences of each OTU to deduce the phylogeny. However, probabilistic techniques such as DADA2 (Callahan et al., 2016) have recently gained more attention, and are now increasingly used to replace the standard OTU clustering approaches by ASVs, which are un-clustered error-corrected reads. Although amplicon sequencing is cost-effective, the reliability of bacterial classification decreases below genus level, and this methodology does not directly quantify bacterial genes and functions.

**Shotgun metagenomics data**. A growing number of studies use shotgun metagenomics and offer untargeted sequence data from the analyzed samples. These data typically include contamination from host or environmental reads as well. The non-host DNA can be used for taxonomic analysis or functional profiling of all types of microorganisms present in the microbiome–it allows the analyses of bacteria, viruses, fungi and parasites at the same time. Sequences from metagenomic data can be classified using existing databases or assembled *de novo*. This type of analysis offers the possibility to analyze strain or even SNP level dynamics of the microbiome (Quince et al., 2017; Zeevi et al., 2019) as well as reconstruction of draft genomes, which enables the identification of novel organisms and provides a way to link functions with taxa. Depending on the aims of the study, shotgun metagenomics can provide a variable amount of data as shallow, deep, or even ultradeep sequencing (Hillmann et al., 2018).

**Metatranscriptome data**. Metatranscriptomics characterize the expressed transcripts of the analyzed community at a given time point/conditions transcripts of the analyzed community by RNA sequencing data. Depending on the sequencing depth, with this method it is possible to obtain information on gene expression levels both for the microbiome communities and for the host. This requires the highest sequencing depth, most stringent standards for sample storage and processing, and data analysis workflows and benchmarking for these data are only in the developmental stage. Despite these advantages, metatranscriptomes will need to be supported by additional shotgun metagenomics measurements for accurate interpretation.

**Other-omics data such as metabolomics data, metaproteomics data**. These data represent directly measured metabolites or expressed proteins, therefore providing additional functional information. Similarly, these data can contain information both from the microbiome and the host.

The special characteristics of metagenomic sequencing data are posing additional challenges for statistical analysis. For instance, the large inter-individual variability, heteroscedastic variation (i.e., variance increasing with mean abundance) and large biological and technical variations are often not properly approximated by classical Gaussian or log-normal models, requiring customized analytical approaches. Microbiome data sets tend to be sparse and skewed, and typically include many more microbial features compared to the number of samples or observations collected in most microbiome studies to date (Supplementary Table S1). Moreover, microbiome features often exhibit complex and hierarchical dependency structures in terms of taxonomies or co-variation in abundance and function. Moreover, unaligned and misaligned sequence reads, and challenges to distinguish technical and biological variation especially at the level of low-abundant organisms add additional challenges to the microbiome analyses. The demand to represent microbiome data with an arbitrary, but fixed sum of components without loss of information are known from the concept of compositional data (Aitchison, 1986; Gloor et al., 2017). Furthermore, complementary multi-omic and other data types (Box 1) may require different modeling approaches. The integration of different types of data often lacks rigorous model selection procedures, correction for multiple testing, handling of missing data features/labels, or data harmonization and integration (Namkung, 2020).

Finally, the reliability and integration of relevant metadata such as demographics, health, diet, age, medication, lifestyle, and other factors are critical for drawing informative insights from microbiome studies. However, these crucial pieces of information are most often missing or insufficiently machine-readable in publicly available data resources, thus forming bottlenecks on data reuse.

### Statistics and Machine Learning Aspects

Microbiome research has set fresh challenges for statistical analysis. Instead of a thorough literature review of this rapidly expanding and heterogeneous field, we provide hereby a topical perspective on the application of ML techniques in microbiome research (for an extensive review, please see Marcos-Zambrano et al., 2021).

One of the most common applications of ML is dimensionality reduction, which facilitates the exploration and visualization of community similarity and distribution across the population of study samples. Non-linear approaches have become a common choice due to the inherent complexity of microbial communities, including methods such as PCoA, UMAP, and other techniques (Legendre and Legendre, 2012; Becht et al., 2019; Kobak and Berens, 2019), as well as autoencoders (Oh and Zhang, 2020) have been taken into use. Many automated analysis pipelines readily include these methods (Buza et al., 2019; Liao et al., 2019).

Clustering has found many applications in microbiome research, ranging from data preprocessing to downstream community analyses. A popular method is the denoiser DADA2 (Callahan et al., 2016), designed to identify unique 16S rRNA amplicon sequence variants (ASVs) (Davis et al., 2018). In metagenome sequencing studies, probabilistic methods have been used to assemble contigs into genome bins based on information of abundance and sequence information; CONCOCT (Alneberg et al., 2014) implements non-parametric clustering based on a variational Gaussian mixture model. The advantage of the non-parametric approach is the automated determination of the cluster number based on the model, rather than *post hoc* evaluation indices such as the Kalinski-Harabasz or Silhouette index. In the downstream analysis of microbiome data, a notable application of clustering algorithms has been the identification of microbiome *community types*, used to stratify individuals into specific subgroups based on microbiome composition (Holmes et al., 2012; Costea et al., 2018). Recently, more detailed assemblage models have been developed to identify latent factors and sub-communities that can complement ecosystem-wide stratification that focuses on overarching community types. Examples include phylofactor (Washburne et al., 2019), tipping elements (Lahti et al., 2014), non-negative matrix factorization, latent Dirichlet allocation, and other latent mixture models (Sankaran and Holmes, 2019).

Classification methods are commonly used in taxonomic assignment of metagenomic reads to annotate genome sequences (Treangen et al., 2013; Tamames et al., 2019) or in the production of metagenome-assembled genomes (Murovec et al., 2020). Another application is sample classification in diagnostic or prognostic studies (Pasolli et al., 2016; Aryal et al., 2020). Common ML algorithms such as random forest, support vector machines (SVM), elastic net, and LASSO have all been used for disease-prediction tasks (Pasolli et al., 2016), and automated feature selection schemes have been reported to perform will in comparison with standard tests in disease prediction (Ai et al., 2017). Instead of hard classification, some applications focus on detecting estimated percentage contribution, or soft classification, of each potential source environment related to the sample (Knights et al., 2011; Shenhav et al., 2019; McGhee et al., 2020).

Deep learning (DL) is increasingly applied in microbiome research Convolutional Neural Networks (CNNs) (Armour et al., 2019) have recently been augmented with phylogenetic tree information (Reiman et al., 2018), or combined neural networks with random forests (Rahman and Rangwala, 2020). Variable evaluation metrics including accuracy, precision, recall, F1-score and area under curve (AUC), have been used, highlighting the need for standardized benchmarks regarding well-defined modeling tasks; systematic evaluations have been carried out for instance for metagenome-based disease prediction and differentiation of body sites based on microbiome composition (Asgari et al., 2018; Reiman et al., 2018; Díez López et al., 2019; LaPierre et al., 2019). DL has been also applied to classify antibiotic resistance genes (ARGs) derived from metagenomic data (Arango-Argoty et al., 2018) and to overcome the lack of well-curated taxonomic trees for newly discovered species in metagenome assembled genomes (Murovec et al., 2020). DL has also been used to predict how gut microbiome responds to perturbations by antibiotics (Rahman et al., 2018). Whereas DL methods are notoriously data-hungry, recent applications have shown promising performance with moderate training sample sizes.

A vast number of microbiome studies quantify associations between the abundances of specific metagenomic and functional features, and key covariates such as health and disease, and other factors including diet, medication, geography, or stool consistency (Turnbaugh et al., 2007; Qin et al., 2010; Falony et al., 2016; Zhernakova et al., 2016). The analysis covers a vast spectrum of standard ML methods with additional adaptations to microbiome data. Popular approaches include adaptations of linear discriminant analysis (Segata et al., 2011), negative binomials (Love et al., 2014), and Dirichlet distributions (Fernandes et al., 2014), and non-parametric methods (Weiss et al., 2017; Lin and Peddada, 2020). Non-parametric regression models, such as Gaussian processes, have been also used to study associations between microbiome diversity and external conditions (Arbel et al., 2016). Common techniques for community comparisons include regularized discriminant analysis (RDA) (Legendre and Legendre, 2012), random forest (Sze and Schloss, 2018; Topçuoğlu et al., 2020), and gradient boosting (Qin et al., 2020; Topçuoğlu et al., 2020). Further strategies have been developed in order to consider hierarchical dependencies between taxonomic groups to control for multiple testing and to identify the appropriate taxonomic levels for associations (Sankaran and Holmes, 2014; Washburne et al., 2017).

Other emerging applications include spatio-temporal modeling of microbiome variation both at the individual and population levels as well as the biogeographical variation within and across body sites; agent-based models provide interesting opportunities in this area (Juhász et al., 2014; Lin et al., 2018). Probabilistic joint species distribution models have also been recently applied in the microbiome context (Björk et al., 2018). Bayesian ML techniques can help to deal with uncertainties related to the limited information in short and sparse time series or spatial sampling. The uncertainty, the limited sampling density, or the limited amount of labeled examples when training a model can also be addressed through semi-supervised methods. Prospective analyses predicting long-term incident of health and disease risk based on microbiome composition have remained scarce due to the lack of large-scale cohorts with long-term follow-ups, but the need for prospective analysis methods is now emerging (Liu et al., 2020; Salosensaari et al., 2020). Mendelian randomization and related techniques are finding applications to understand the causal role of gut microbiome in disease (Sanna et al., 2019; Hughes et al., 2020).

## Discussion

Statistics and ML provide tools to extract useful information from scarce, noisy, and limited data. In particular, within microbiome data, this has to be balanced with the complexity and limited understanding of the host-regulated ecological processes and the high levels of individual variation. ML has great potential to improve disease diagnosis and identify personalized biomarkers, due to its ability to detect informative patterns in the data with limited prior knowledge of the underlying system.

One of the main shortcomings is, however, the use of inappropriately small datasets, as apparent from the example studies (and their corresponding datasets) listed in Supplementary Table S1. Data accumulation will further enhance the use of more advanced ML technologies. Efficient data structures and making microbiome data Findable, Accessible, Interoperable, and Reusable (FAIR)^1^ can provide invaluable support for the open development of statistical and ML tools to help to advance the field (Shetty and Lahti, 2019). Consequently, data repositories maintained by large consortia could serve as a central resource for the research community (Meyer et al., 2008; Mitchell et al., 2020). However, to this aim, the submission of the metadata must follow controlled vocabulary and minimal standards (ten Hoopen et al., 2017).

Some of the main challenges in detecting associations between specific microbiome features and key covariates are related to choosing appropriate distributional assumptions including sparsity and compositionality, appropriate feature selection, controlling for technical biases such as read count variations, the potential confounding effects, and multiple testing. Successful solutions often present combinations of statistical techniques that have been specifically tailored to fit the particular characteristics of microbiome data. Besides, over-fitting, incomplete model selection or performance assessment can lead to poor generalizability of the results in previously unseen data sets and lack of reproducibility. It is essential to understand the principles underlying each method and follow the recommended guidelines in order to ensure compliance with the modeling assumptions (Rule et al., 2019) and avoid overfitting (Eetemadi et al., 2020). Another important driver for the field is the development of suitable data structures in statistical programming languages, such as the R/Bioconductor ecosystem as curatedMetagenomicData (Pasolli et al., 2016) and the *phyloseq* (McMurdie and Holmes, 2013) or *TreeSummarizedExperiment* classes (Huang et al., 2020), that permit standardization and efficient collaborative development of methods.

The microbiome field is moving from associations to causality, mechanisms, and prediction, and ML will aid in this transition. Data obtained from ML methods can help to propose new hypotheses to be tested in experimental models, as well as to accelerate the translation of the microbiome data into clinical practice. Its optimal use will presumably trigger the improvement of the searching of biomarker candidates for disease diagnostics, prognostics, and the use of statistical inference for causal insights (Pearl, 2009; Walhout et al., 2013), as with the increasing need to model temporal and dynamical variation. But these advances will appear through validation of the results obtained by sequencing (e.g., using an independent approach such as qPCR), followed by combinations with other omics, especially with metabolomics and metatranscriptomics.

Interpretability by non-experts is an essential consideration when ML models are put in practice by translational researchers. To overcome existing trade-offs between model interpretability and performance (Topçuoğlu et al., 2020) an active collaboration and joint education/training of researchers from statistical, biomedical and clinical fields is essential. Therefore, one main priority is the development of user-friendly tools for translational and clinical personnel, who may have limited experience with bioinformatics methods. In this line, popular software like mothur (Schloss et al., 2009, QIIME2 (Bolyen et al., 2019), and MicrobiomeAnalyst (Chong et al., 2020), the R/Bioconductor ecosystem (Qin et al., 2010), Anvi’o (Eren et al., 2015), and Biobakery (McIver et al., 2018) have incorporated ML methods into their applications in a readily usable format. Hence, the role of open source software ecosystems is critical for the overall development of the whole field. This can support and advance open collaboration networks and co-creation models that have been further complemented with open benchmark data sets (Olson et al., 2017) and reproducible notebooks (Rule et al., 2019). None of the above, however, can be achieved without multidisciplinary training of “next-generation” experts that could be integrated in clinical environments, ultimately facilitating clinical decision-making based on microbiome data as part of personalized medicine strategies (Gómez-López et al., 2019).

**FIGURE 1:**
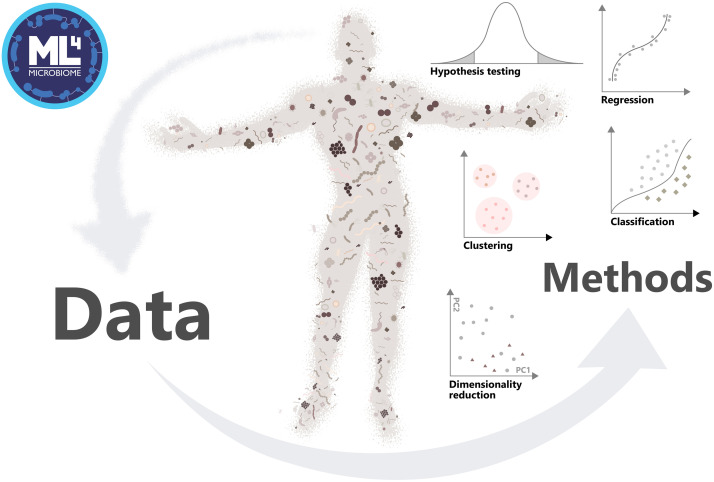
Approach of the Action ML4Microbiome to the implementation of machine learning methods in microbiome research, driving change of the field in personalized medicine.

In order to accelerate this transition, the COST (European Cooperation in Science and Technology) Action “ML4Microbiome” (Machine Learning for Microbiome) started in 2019 with the aim to coordinate a synergistic network of the use of ML in Microbiome research at the European level. This COST Action CA18131 on *Statistical and Machine Learning Techniques in Human Microbiome Studies* is a step toward tackling the challenges by strengthening the network of European researchers in this emerging research area (Figure 1). A space of discussion to break down barriers of communication between fields, as well as their engagement, is being constructed through its four working groups (WG) and several networking and training events http://www.ml4microbiome.eu. It is also planned to launch a DREAM challenge^2^. DREAM challenges are crowdsourced benchmark efforts. By decoupling the method development (open to any scientist) to their evalution (by the organizers based on hold-back data, these challenges provide an unbiased and transparent assessment of methods (Saez-Rodriguez et al., 2016). Furthermore, the action ML4Microbiome identified multiple shortcomings in the current research that need to be taken into consideration. The field will benefit from increasing sample sizes, and the availability of spatial and longitudinal profiling that can be used to train more detailed and accurate models of microbiome variation. The development of interpretable and transparent ML methods will help to bridge the gap between methodological and applied fields. ML4Microbiome is open for new multi-disciplinary collaborations and collaborative ML methods development, and is welcoming researchers to participate in workshops, courses, source code/tool development aiming to promote the use of appropriate statistical and machine learning methods in metagenomics.

## Data Availability Statement

The original contributions presented in the study are included in the article/Supplementary Material, further inquiries can be directed to the corresponding author/s.

## Author Contributions

IM-I, AZ, DG-C, and MC conceived the manuscript. IM-I, LL, MN, IE, and GR coordinated, supervised, and wrote the draft, the Supplementary Information, and the final manuscript. MA, OA, BB-G, ES, DD’E, MD, LF, AG, KH, TK, ML, LM-Z, CM, MM, PM, LP, GP, SP, VP, PP, AS, RSh, BS, RSu, JT, C-OT, BV, DV, EY, GZ, JS-R, AZ, DG-C, and MC revised draft manuscript, provided comments, included manual references, and wrote parts of the final manuscript. All the authors discussed and approved the final version of the manuscript.

## Conflict of Interest

The authors declare that the research was conducted in the absence of any commercial or financial relationships that could be construed as a potential conflict of interest.

## Footnotes

1 https://microbiomedata.org/fair/

2 www.dreamchallenges.org

## Supplementary Material

Supplementary Table 1 Summary and main characteristics of human microbiome studies employing ML approaches.
